# Progress Toward Measles Elimination — African Region, 2013–2016

**DOI:** 10.15585/mmwr.mm6617a2

**Published:** 2017-05-05

**Authors:** Balcha G. Masresha, Meredith G. Dixon, Jennifer L. Kriss, Reggis Katsande, Messeret E. Shibeshi, Richard Luce, Amadou Fall, Annick R.G.A. Dosseh, Charles R. Byabamazima, Alya J. Dabbagh, James L. Goodson, Richard Mihigo

**Affiliations:** ^1^Immunization and Vaccines Development Program, World Health Organization (WHO), Regional Office for Africa, Brazzaville, Congo; ^2^Global Immunization Division, Center for Global Health, CDC; ^3^Expanded Program on Immunization, WHO Regional Office for Africa, Inter-Country Support Team, Harare, Zimbabwe; ^4^Expanded Program on Immunization, WHO Regional Office for Africa, Inter-Country Support Team, Libreville, Gabon; ^5^Expanded Program on Immunization, WHO Regional Office for Africa, Inter-Country Support Team, Ouagadougou, Burkina Faso; ^6^Department of Immunization, Vaccines, and Biologicals, WHO, Geneva, Switzerland.

In 2011, the 46 World Health Organization (WHO) African Region (AFR) member states established a goal of measles elimination* by 2020, by achieving 1) ≥95% coverage of their target populations with the first dose of measles-containing vaccine (MCV1) at national and district levels; 2) ≥95% coverage with measles-containing vaccine (MCV) per district during supplemental immunization activities (SIAs); and 3) confirmed measles incidence of <1 case per 1 million population in all countries (*1*). Two key surveillance performance indicator targets include 1) investigating ≥2 cases of nonmeasles febrile rash illness per 100,000 population annually, and 2) obtaining a blood specimen from ≥1 suspected measles case in ≥80% of districts annually (*2*). This report updates the previous report (*3*) and describes progress toward measles elimination in AFR during 2013–2016. Estimated regional MCV1 coverage^†^ increased from 71% in 2013 to 74% in 2015.^§^ Seven (15%) countries achieved ≥95% MCV1 coverage in 2015.^¶^ The number of countries providing a routine second MCV dose (MCV2) increased from 11 (24%) in 2013 to 23 (49%) in 2015. Forty-one (79%) of 52 SIAs** during 2013–2016 reported ≥95% coverage. Both surveillance targets were met in 19 (40%) countries in 2016. Confirmed measles incidence in AFR decreased from 76.3 per 1 million population to 27.9 during 2013–2016. To eliminate measles by 2020, AFR countries and partners need to 1) achieve ≥95% 2-dose MCV coverage through improved immunization services, including second dose (MCV2) introduction; 2) improve SIA quality by preparing 12–15 months in advance, and using readiness, intra-SIA, and post-SIA assessment tools; 3) fully implement elimination-standard surveillance^††^; 4) conduct annual district-level risk assessments; and 5) establish national committees and a regional commission for the verification of measles elimination.

## Immunization Activities

WHO and the United Nations Children’s Fund (UNICEF) estimate vaccination coverage using annual government-reported administrative data and data from independent surveys. During 2013–2015, the estimated MCV1 coverage in AFR increased from 71% to 74%, while the number of AFR countries with ≥95% MCV1 coverage decreased from eight (17%) to seven (15%) (Table 1). In 2015, national MCV1 coverage was highest in Mauritius (99%), Tanzania (99%), and Seychelles (98%), and lowest in South Sudan (20%), Equatorial Guinea (27%), and the Central African Republic (49%). The number of countries providing a routine MCV2 dose increased from 11 (24%) in 2013 to 23 (49%) in 2015. Estimated regional MCV2 coverage increased from 7% in 2013 to 18% in 2015. During 2013–2016, approximately 300 million children received MCV during 52 SIAs conducted in 42 (89%) countries (Table 2). In 41 (79%) SIAs, reported administrative coverage was ≥95%. Among 25 (48%) SIAs for which a post-SIA coverage survey was conducted, estimated coverage of ≥95% was achieved in eight (32%).

**TABLE 1:** Estimated coverage with the first dose (MCV1)* and second dose (MCV2)*^,†^ of measles-containing vaccine, number of confirmed measles cases,^§^ and confirmed measles incidence per 1 million population,^¶^ by country — World Health Organization (WHO) African Region, 2013–2016

Country	2013			2014			2015			2016
	Coverage (%)		No. of confirmed cases^§^ (incidence^¶^)	Coverage (%)		No. of confirmed cases^§^ (incidence^¶^)	Coverage (%)		No. of confirmed cases^§^ (incidence^¶^)	No. of confirmed cases^§^ (incidence^¶^)
	MCV1	MCV2^†^		MCV1	MCV2^†^		MCV1	MCV2^†^		
Algeria	95	93	0 (0.0)	95	99	0 (0.0)	95	99	62 (1.6)	27 (0.7)
Angola	66	—	6,297 (268.5)	60	—	11,648 (480.8)	55	26	67 (2.7)	33 (1.3)
Benin	68	—	735 (71.2)	68	—	768 (72.5)	75	—	53 (4.9)	90 (8.1)
Botswana	97	83	1 (0.5)	97	85	88 (39.6)	97	85	2 0.9)	1 (0.4)
Burkina Faso	82	—	431 (25.2)	88	17	433 (24.6)	88	50	99 (5.5)	222 (11.9)
Burundi	98	51	0 (0.0)	94	60	5 (0.5)	93	65	9 (0.8)	17 (1.5)
Cameroon	83	—	766 (34.5)	80	—	720 (31.6)	79	—	1,785 (76.5)	324 (13.5)
Cape Verde	91	89	0 (0.0)	93	79	0 (0.0)	92	95	0 (0.0)	0 (0.0)
Central African Republic	25	—	370 (78.5)	49	—	212 (44.1)	49	—	147 (30.0)	156 (31.2)
Chad	59	—	185 (14.1)	54	—	1,237 (91.0)	62	—	435 (31.0)	147 (10.1)
Comoros	82	—	0 (0.0)	80	—	0 (0.0)	81	—	0 (0.0)	0 (0.0)
Congo	80	—	123 (28.0)	80	—	70 (15.5)	80	—	1,358 (293.9)	292 (61.6)
Cote d'Ivoire	76	—	48 (2.2)	62	—	50 (2.3)	72	—	40 (1.8)	52 (2.2)
Democratic Republic of Congo	76	—	2,470 (34.0)	77	—	1,595 (21.3)	79	—	4,471 (57.9)	4,790 (60.1)
Equatorial Guinea	42	—	6 (7.5)	44	—	9 (11.0)	27	—	1,232 (1,457.9)	1,685 (1,937.7)
Eritrea	94	—	47 (9.4)	90	—	1 (0.2)	85	75	91 (17.4)	59 (11.0)
Ethiopia	62	—	6,029 (63.8)	70	—	12,485 (128.8)	78	—	16,123 (162.2)	4,484 (44.0)
Gabon	70	—	127 (77.0)	61	—	42 (24.9)	68	—	37 (21.4)	1,274 (722.6)
Gambia	96	53	1 (0.5)	96	73	2 (1.0)	97	77	21 (10.5)	40 (19.5)
Ghana	89	54	318 (12.2)	92	67	143 (5.3)	89	63	51 (1.9)	53 (1.9)
Guinea	62	—	39 (3.3)	52	—	35 (2.9)	52	—	29 (2.3)	130 (10.0)
Guinea-Bissau	69	—	0 (0.0)	69	—	0 (0.0)	69	—	0 (0.0)	0 (0.0)
Kenya	73	—	215 (4.9)	79	—	356 (7.9)	75	28	110 (2.4)	61 (1.3)
Lesotho	90	82	2 (1.0)	90	82	4 (1.9)	90	82	2 (0.9)	13 (6.0)
Liberia	74	—	0 (0.0)	58	—	0 (0.0)	64	—	433 (96.1)	391 (84.7)
Madagascar	63	—	8 (0.3)	64	—	3 (0.1)	58	—	7 (0.3)	22 (0.9)
Malawi	88	—	1 (0.1)	85	—	2 (0.1)	87	8	19 (1.1)	4 (0.2)
Mali	80	—	308 (18.6)	80	—	274 (16.0)	76	—	240 (13.6)	107 (5.9)
Mauritania	80	—	3 (0.8)	84	—	14 (3.5)	70	—	1 (0.2)	13 (3.1)
Mauritius	99	85	0 (0.0)	98	85	0 (0.0)	99	85	0 (0.0)	0 (0.0)
Mozambique	85	—	57 (2.2)	85	—	80 (2.9)	85	—	78 (2.8)	84 (2.9)
Namibia	82	—	495 (210.9)	83	—	718 (298.8)	85	—	216 (87.8)	13 (5.2)
Niger	76	—	790 (43.0)	72	3	294 (15.4)	73	16	603 (30.3)	591 (28.5)
Nigeria	47	—	50,585 (292.7)	51	—	4,470 (25.2)	54	—	11,494 (63.1)	11,499 (61.5)
Rwanda	95	—	17 (1.5)	97	—	5 (0.4)	97	87	1 (0.1)	57 (4.8)
Sao Tome and Principe	91	—	0 (0.0)	92	71	0 (0.0)	93	76	0 (0.0)	0 (0.0)
Senegal	84	—	13 (0.9)	80	13	38 (2.6)	80	54	58 (3.8)	159 (10.2)
Seychelles	97	97	0 (0.0)	99	98	0 (0.0)	98	98	0 (0.0)	0 (0.0)
Sierra Leone	83	—	13 (2.1)	78	—	44 (7.0)	76	60	139 (21.5)	195 (29.6)
South Africa	66	53	61 (1.1)	70	60	98 (1.8)	76	63	18 (0.3)	24 (0.4)
South Sudan	30	—	0 (0.0)	22	—	0 (0.0)	20	—	341 (27.6)	845 (66.4)
Swaziland	85	89	0 (0.0)	86	89	0 (0.0)	78	89	0 (0.0)	1 (0.8)
Tanzania	99	—	191 (3.8)	99	29	61 (1.2)	99	57	19 (0.4)	36 (0.7)
Togo	72	—	321 (46.3)	82	—	168 (23.6)	85	—	21 (2.9)	29 (3.9)
Uganda	82	—	452 (12.4)	82	—	313 (8.3)	82	—	478 (12.2)	250 (6.2)
Zambia	80	—	1 (0.1)	85	33	16 (1.0)	90	47	20 (1.2)	7 (0.4)
Zimbabwe	93	—	3 (0.2)	92	—	65 (4.3)	86	—	1 (0.1)	2 (0.1)
**African Region**	**71**	**7**	**71,529** (**76.3**)	**72**	**11**	**36,566** (**38.0**)	**74**	**18**	**40,411** (**40.9**)	**28,279** (**27.9**)

* WHO-United Nations Children’s Fund (UNICEF) estimate.

^†^ Cells containing “—“ indicate that the corresponding country has not yet introduced MCV2.

^§^ Measles case-based surveillance. Confirmed cases were defined by laboratory criteria, epidemiologic linkage, or clinical criteria. Laboratory-confirmed was defined as having a measles-specific immunoglobulin M–positive test result and not receiving a measles vaccination during the 30 days before rash onset. Epidemiologically linked was defined as meeting the suspected measles case definition and having contact (i.e., lived in the same district or an adjacent district, with plausibility of transmission) with a patient with a laboratory-confirmed measles case with rash onset within the preceding 30 days. Clinically compatible was defined as meeting the case definition for measles, with no specimen available for laboratory testing and no evidence of epidemiologic linkage to a laboratory-confirmed case. A suspected measles case was defined as an illness characterized by rash, fever, and one or more of the following symptoms: conjunctivitis, coryza, or cough, or an illness in any patient in whom the clinician suspected measles.

^¶^ Incidence per 1 million population was calculated using the United Nations Population Division World Population Prospects: 2015 revision.

**TABLE 2:** Characteristics of national and subnational measles supplementary immunization activities (SIAs),*^,†,§^ by year and country — World Health Organization African Region, 2013–2016

Year	Country	Type of SIA*	Age group targeted	Extent of SIA	Children reached in target age group		% of districts with ≥95% administrative coverage^¶,^**	Estimated SIA coverage by survey (%)**
					No.	Administrative coverage (%)^†,¶^		
2013	Botswana	Follow-up M	9–59 m	N	198,341	95	54	—
2013	Cape Verde	Catch-up MR	9 m–24 y	N	240,166	95	46	—
2013	Comoros	Follow-up M	6–59 m	N	86,516	86	59	93
2013	Congo	Follow-up M	6–59 m	N	726,979	92	58	86
2013	Democratic Republic of the Congo	Follow-up M	6 m–9 y	SN	11,019,958	100	—	—
2013	Ethiopia	Follow-up M	9–59 m	N	11,608,063	99	66	91
2013	Ghana	Catch-up MR	9 m–14 y	N	11,062,605	99	70	96
2013	Lesotho	Follow-up M	9–59 m	N	147,676	73	90	92
2013	Madagascar	Follow-up M	9–59 m	N	3,316,542	92	56	84
2013	Malawi	Follow-up M	9–59 m	N	2,405,108	105	100	96
2013	Mozambique	Follow-up M	9–59 m	N	4,078,637	102	95	81
2013	Nigeria	Follow-up M	9–59 m	SN	30,579,666	103	—	75
2013	Rwanda	Catch-up MR	9 m–14 y	N	4,391,081	103	90	98
2013	Senegal	Catch-up MR	9 m–14 y	N	6,097,155	101	76	97
2013	South Africa	Follow-up M	6–59 m	N	4,186,191	100	60	—
2013	Swaziland	Follow-up M	6–59 m	N	119,207	97	—	91
2013	Togo	Follow-up M	9 m–9 y	N	1,641,635	96	83	—
2014	Angola	Follow-up M	6 m–9 y	N	7,829,940	117	84	97
2014	Benin	Follow-up M	9 m–9 y	N	3,009,405	101	82	97
2014	Burkina Faso	Catch-up MR	9 m–14 y	N	8,517,508	107	100	—
2014	Chad	Follow-up M	6 m–9 y	SN	2,549,188	103	94	—
2014	Côte d’Ivoire	Follow-up M	6 m–9 y	N	9,640,512	92	95	95
2014	Democratic Republic of Congo	Follow-up M	6 m–9 y	SN	20,699,401	101	87	—
2014	Mauritania	Follow-up M	9 m–14 y	N	1,489,563	105	92	—
2014	South Sudan	Follow-up M	6–59 m	N	1,715,139	122	98	77
2014	Tanzania	Catch-up MR	9 m–14 y	N	20,529,629	97	59	89
2015	Benin	Follow-up M	9 m–9 y	N	408,511	102	—	—
2015	Cameroon	Catch-up MR	9 m–14 y	N	9,229,739	98	80	89
2015	Eritrea	Follow-up M	9–59 m	N	350,765	80	36	—
2015	Guinea-Bissau	Follow-up M	9–59 m	N	223,673	86	18	—
2015	Liberia	Follow-up M	6–59 m	N	596,545	99	80	90
2015	Mali	Follow-up M	9 m–14 y	N	9,312,619	112	91	94
2015	Niger	Follow-up M	9–59 m	N	3,299,923	96	75	—
2015	Nigeria	Follow-up M	9–59 m	N	43,134,811	110	88	85
2015	Sierra Leone	Follow-up M	9–59 m	N	1,205,865	97	71	—
2015	Togo	Follow-up M	9 m–9 y	SN	820,335	99	94	—
2015	Uganda	Follow-up M	6–59 m	N	6,349,182	95	56	—
2015	Zimbabwe	Catch-up MR	9 m–14 y	N	5,337,029	103	100	94
2016	Botswana	Catch-up MR	9 m–14 y	N	674,150	95	67	—
2016	Central African Republic	Follow-up M	6–59 m	N	1,529,441	84	20	—
2016	Chad	Follow-up M	6–59 m	N	2,342,341	112	99	—
2016	Comoros	Follow-up M	6–59 m	N	80,614	74	41	—
2016	Democratic Republic of Congo	Follow-up M	6–59 m	N	10,921,820	101	93	—
2016	Equatorial Guinea	Follow-up M	6–59 m	N	127,874	85	61	—
2016	Gambia	Catch-up MR	9 m–14 y	N	779,654	97	86	—
2016	Guinea	Follow-up M	9–59 m	N	2,412,923	103	94.7	92.7
2016	Kenya	Catch-up MR	9 m–14 y	N	19,154,577	101	77	95
2016	Madagascar	Follow-up M	9–59 m	N	3,547,456	95	75	—
2016	Namibia	Catch-up MR	9 m–39 y	N	1,908,193	103	77	—
2016	Sao Tome and Principe	Catch-up MR	9 m–14 y	N	77,285	107	100	—
2016	Swaziland	Catch-up MR	9 m–14 y	N	373,508	90	—	94
2016	Zambia	Catch-up MR	9 m–14 y	N	7,741,505	108	97	—
**TOTAL**	—	—	—	—	**299,826,149**	**102**	—	—

**Abbreviations:** M = measles vaccination; MR = measles-rubella vaccination; m = months; N = national; SN = subnational; y = years.

* SIAs generally are carried out using two target age ranges. An initial, nationwide catch-up SIA focuses on all children aged 9 months–14 years, with the goal of eliminating susceptibility to measles in the general population. Periodic follow-up SIAs then focus on all children born since the last SIA. Follow-up SIAs generally are conducted nationwide every 2–4 years, depending on routine immunization coverage, and focus on children aged 9–59 months; their goal is to eliminate any measles susceptibility that has developed in recent birth cohorts and to protect children who did not respond to the first dose of measles-containing vaccine. The target age range for follow-up SIAs might be widened to include older children based on the measles susceptibility pattern in countries. Countries introducing rubella vaccine do so via wide age-range combined measles-rubella vaccine campaigns.

^†^ Data source is the World Health Organization, African Region. Data were last updated March 10, 2017.

^§^ This table excludes seven outbreak response immunization campaigns that occurred in five countries (Ethiopia, Guinea, Malawi, Sierra Leone, and South Sudan) and which vaccinated approximately 40.4 million children.

^¶^ Administrative coverage is defined as the number of vaccine doses provided divided by the total number of children in the age group targeted, multiplied by 100.

** Cells containing “— “ indicate that data was not available at time of publication or that no coverage survey was performed.

## Surveillance Activities

Countries performing measles case-based surveillance electronically report surveillance data^§§^ weekly to the WHO AFR office. Measles case-based surveillance involves completing a case investigation form^¶¶^ and collecting a blood specimen for laboratory testing (*2*). Suspected measles cases are confirmed by laboratory testing, epidemiologic linkage to a confirmed case, or by clinical criteria.*** During 2013–2016, all but three AFR countries^†††^ conducted case-based surveillance with access to standardized quality-controlled testing at 47 laboratories within the WHO Global Measles and Rubella Laboratory Network^§§§^ (*4*). During 2013–2016, the number of countries that met both surveillance targets (i.e., investigated two or more cases of nonmeasles febrile rash illness per 100,000 population annually and obtained a blood specimen from at least one suspected measles case in ≥80% of districts) (19 countries), one of the surveillance targets (12), and neither surveillance target (16) remained stable (Figure). Although the total number of countries per category remained constant, performance declined in seven (15%) countries, improved in nine (19%), and was unchanged in 31 (66%).

**FIGURE:**
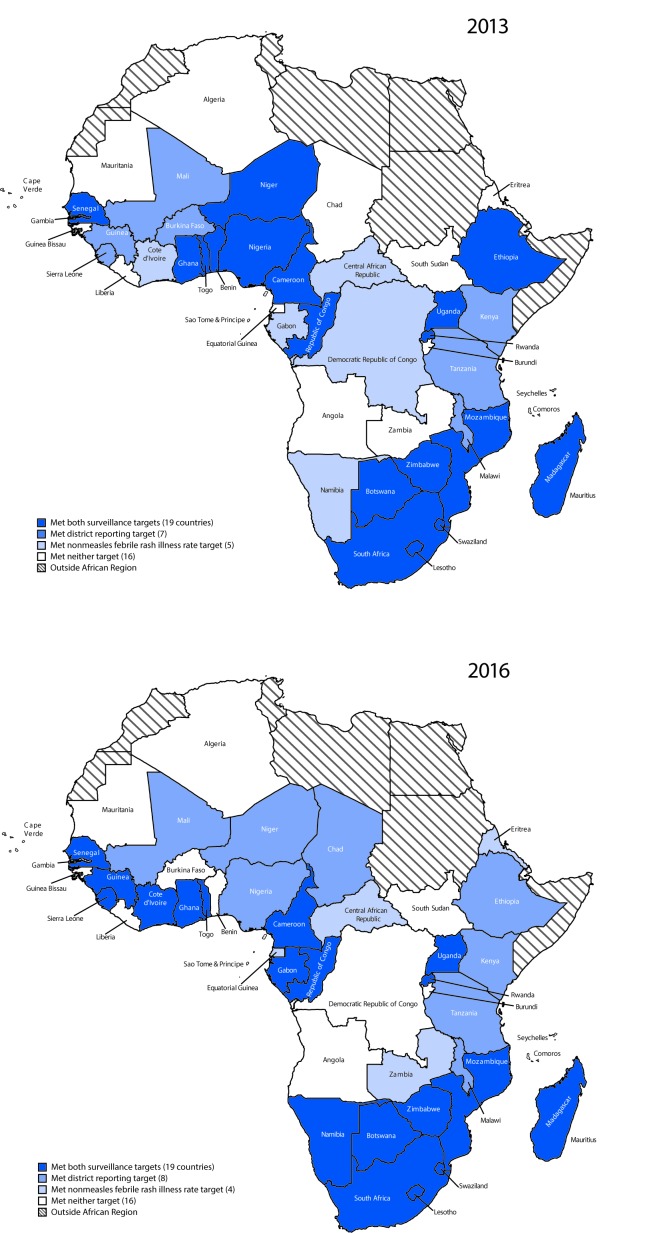
Measles case-based surveillance performance* by country — World Health Organization African Region, 2013 and 2016 * Two key surveillance performance indicator targets were 1) investigate ≥2 cases of nonmeasles febrile rash illness per 100,000 population annually (nonmeasles febrile rash illness rate target), and 2) obtain a blood specimen from ≥1 suspected measles case in ≥80% of districts annually (district reporting target).

## Disease Incidence

Overall, 176,785 confirmed measles cases were reported in AFR through case-based surveillance during 2013–2016 (Table 1). The number of confirmed measles cases declined 60%, from 71,529 in 2013 to 28,279 in 2016. During 2013–2016, a total of 103,161 (60%) reported measles cases occurred among children aged 9–59 months, 79% of whom were either unvaccinated or had unknown vaccination status. Confirmed measles incidence decreased 63% from 76.3 per 1 million population in 2013 to 27.9 in 2016 (Table 1). The largest percentage decreases in incidence occurred in Angola (99%), Namibia (97%), and Togo (92%). The highest confirmed measles incidences in 2016 were reported in Equatorial Guinea (1,938 per 1 million), Gabon (723), and Liberia (85). The number of countries that reported less than one case per 1 million population decreased from 19 (41%) to 15 (32%). During 2013–2016, 249 measles virus genotype results were reported from 14 (30%) countries; all were genotype B3.

## Discussion

Although measles incidence decreased 63% in AFR during 2013–2016, the region did not meet vaccination coverage, surveillance, and disease incidence targets needed to achieve measles elimination by 2020. During 2013–2015, estimated MCV1 coverage increased only 3%, and in 2015 was <95% in 87% of AFR countries. Among the estimated 8.9 million infants in AFR who did not receive MCV1 in 2015, approximately 4.8 million (54%) resided in Nigeria (3 million), Ethiopia (0.7 million), the Democratic Republic of the Congo (DRC) (0.6 million), and Angola (0.5 million) (*4*). WHO recommends that all countries include a second routine dose of MCV in their national vaccination schedules, irrespective of the level of MCV1 coverage (*5*); only half of all AFR countries have done so. Eliminating the previous stringent MCV1 coverage requirement^¶¶¶^ allows all countries to introduce MCV2 and establish a well-child visit during the second year of life, providing a timely catch-up opportunity for children missing MCV1 or other vaccines (*6*). WHO advises continuation of national follow-up SIAs until high population immunity (≥93%–95% coverage) is achieved and sustained in all districts with a routine 2-dose MCV schedule (*5*).

During 2013–2016, only 32% of 25 SIAs where a postcampaign survey was conducted had estimated coverage ≥95%, although >100% administrative coverage was reported by nearly half of all 52 SIAs. To achieve SIA coverage targets, WHO SIA guidelines and tools**** should be used to prepare and implement high-quality campaigns, which are subsequently evaluated by coverage surveys. SIA planning should begin 12–15 months before the SIA, and intra-SIA and post-SIA monitoring should be performed to identify low MCV coverage areas so that vaccination of children missed during the SIA can be arranged.

Nearly two-thirds of countries did not attain surveillance indicator targets in 2016, and 15% of countries had poorer surveillance performance in 2016 than in 2013. Fifteen (32%) countries achieved the target of <1 case per 1 million population in 2016. However, most confirmed cases detected during 2013–2016 were among children aged 9–59 months who were unvaccinated or had unknown vaccination status. In addition, 84% of cases were reported from the same four countries that accounted for half of children who missed MCV1: Nigeria (44%), Ethiopia (22%), Angola (10%), and DRC (8%). The recent WHO Measles and Rubella Global Strategic Plan Midterm Review emphasized the limits of MCV coverage data as an indicator and recommended, with SAGE endorsement, using measles disease incidence as another indicator to guide elimination efforts (*7*). To measure measles incidence accurately, however, high-quality, case-based surveillance is crucial; this requires increasing resources for full implementation, particularly as countries transition polio eradication resources to other public health priorities.

The findings in this report are subject to at least two limitations. First, vaccination coverage data can be either incorrectly high or low because of inaccurate target population size estimates, erroneous reporting of doses delivered, and inclusion of SIA doses administered to children outside the target age group. Second, surveillance data underestimate the actual number of cases because not all patients with measles seek care, and not all of those seeking care are reported. In 2016, large discrepancies in the number of case-based and aggregate reported measles cases existed, particularly in DRC.^††††^ Integrated Disease Surveillance and Response system reports of aggregate measles cases in AFR have historically included more measles cases than those reported through case-based surveillance (*3*). In addition, reported suspected measles cases without confirmatory laboratory testing might actually be rubella cases. Underreporting of measles through case-based surveillance markedly limits case characteristic analysis to guide programs. Strengthening of reporting through case-based surveillance systems is needed to provide more robust data.

To eliminate measles by 2020, AFR countries need to introduce MCV2 and increase coverage through immunization services by better managing human and financial resources, enhancing capacity of health staff for improved access, and increasing demand with community-linked immunization services. SIA quality can be improved through country ownership and SIA preparation starting 12–15 months in advance. Fully implementing laboratory-supported case-based surveillance that meets standards for elimination will require human and financial resources. Annual risk assessments using the WHO programmatic measles risk assessment tool^§§§§^ are necessary to identify districts needing surveillance and programmatic strengthening (*8*). As 2020 approaches, a next step will be to establish national verification committees and a regional commission for the verification of measles elimination (*9*) that can review and document progress toward measles elimination and provide supportive oversight and advocacy for elimination efforts in AFR.

**Summary**

**What is already known about this topic?**

In 2012, the World Health Organization (WHO) and United Nations Children’s Fund (UNICEF) estimated first dose of measles-containing vaccine (MCV1) coverage in countries of the WHO African Region (AFR) to be 73% and >90% in 13 (28%) of 46 AFR countries. Among 35 measles supplementary immunization activities (SIAs) conducted during 2011–2012, 23 (66%) had >95% administrative coverage. Nineteen (44%) countries met the two key surveillance performance indicator targets. In 2012, only 16 (37%) countries met the incidence target of <5 cases per 1 million population.

**What is added by this report?**

In 2015, WHO-UNICEF estimated MCV1 coverage in AFR to be 74%; seven (15%) countries reported ≥95% MCV1 coverage. Among 52 measles SIAs conducted during 2013–2016, 41 (79%) reported ≥95% administrative coverage. In 2016, 19 (40%) countries met both surveillance performance indicator targets. In 2016, only 15 (32%) countries met the target of <1 case per 1 million population.

**What are the implications for public health practice?**

To eliminate measles by 2020, AFR countries need to achieve high (95%) 2-dose measles vaccination coverage, through introduction of a second MCV dose into routine immunization programs, increasing routine immunization coverage, improving SIA quality, fully implementing elimination-standard surveillance, conducting annual district-level risk assessments, and establishing national verification committees and a regional commission for the verification of measles elimination.

## Footnotes

* Measles elimination is defined as the absence of endemic measles virus transmission in a defined geographic area (e.g., region or country) for ≥12 months in the presence of a well performing surveillance system. Regional verification of measles elimination takes place after 36 months of interrupted endemic measles virus transmission. More information is available in the WHO Framework for Elimination of Measles and Rubella published in 2013 (http://www.who.int/wer/2013/wer8809.pdf?ua=1).

† http://www.who.int/immunization_monitoring/routine/immunization_coverage/en/index4.htm.

§ As of March 31, 2017, coverage estimates were not yet available for 2016; thus, the coverage estimates for 2015 were used in this analysis.

¶ The number of countries used in the denominator is 46 for 2013 and 47 for 2014–2016. South Sudan did not join the World Health Organization (WHO) African Region (AFR) until late 2013 and was not included in the count for 2013.

** Supplemental immunization activities (SIAs) are carried out using two target age ranges. An initial, nationwide catch-up SIA focuses on all children aged 9 months–14 years, with the goal of eliminating susceptibility to measles in the general population. Periodic follow-up SIAs then focus on all children born since the last SIA. Follow-up SIAs generally are conducted nationwide every 2–4 years, depending on routine immunization coverage, and focus on children aged 9–59 months; their goal is to eliminate any measles susceptibility that has developed in recent birth cohorts and to protect children who did not respond to MCV1. The target age range for follow-up SIAs might be widened to include older children based on the measles susceptibility pattern in countries. Administrative coverage is calculated as the number of vaccine doses provided divided by the total number of children in the age group targeted, multiplied by 100.

†† The 2015 WHO African Regional Guidelines for Measles and Rubella Surveillance recommend that all Member States implement case-based measles surveillance with lab confirmation, and that countries with sustained low incidence of measles implement elimination-standard surveillance. The surveillance system currently used and the indicators utilized to measure performance by 44 of 47 countries in the Africa Region, have been in place since 2002, and are not considered sensitive enough in countries with very low measles incidence nearing measles elimination. However, to move to a more sensitive system of elimination-standard surveillance, the financial and human resources required to investigate and obtain a blood specimen for every suspected case do not currently exist. The indicators used in this analysis are the main measles surveillance indicators.

§§ Case-based surveillance is the collection of epidemiologic information about each individual case; effective case-based measles surveillance includes confirmatory laboratory testing or epidemiologic linkage to a previous, laboratory-confirmed case.

¶¶ For countries implementing elimination-standard case-based surveillance, WHO recommends that information be collected on 12 core variables: name, sex, age/date of birth, date of rash onset, date of notification, date of investigation, date of specimen collection, vaccination status, date of last measles vaccination, district of residence, reporting district, and travel history 7–21 days before date of rash onset.

*** Measles cases are defined as laboratory confirmed, epidemiologically confirmed, or clinically compatible. Laboratory confirmed cases are suspected measles cases with detectable measles virus-specific immunoglobulin class M antibodies, or from whom measles virus can be isolated or measles virus RNA can be detected in appropriate clinical specimens by a proficient laboratory. Epidemiologically linked confirmed measles cases are those suspected measles cases that have not been confirmed by a laboratory but are geographically and temporally related to a laboratory-confirmed case or, in the event of a chain of transmission, to another epidemiologically confirmed measles case, with dates of rash onset occurring 7–21 days apart. Clinically compatible measles cases are a suspected measles cases with fever and maculopapular rash and at least one of the following: cough, coryza, or conjunctivitis, for which no adequate clinical specimen was collected and which have not been linked epidemiologically to a laboratory-confirmed case of measles or to laboratory-confirmed case of another communicable disease.

††† Mauritius, Sao Tome and Principe, and Seychelles performed clinical surveillance, which entails notifying and reporting suspected cases using symptom-based case definitions, without any laboratory testing, to the national level in a timely manner. Clinical surveillance is performed because these three countries did not have national laboratories for measles testing. These countries did not report through the AFR case-based surveillance system for 2013–2106. WHO is supporting these countries to establish national serologic laboratories.

§§§ The WHO Global Measles and Rubella Laboratory Network supports standardized methods and quality assurance measures in national laboratories across countries, as well as in three regional reference laboratories (Abidjan, Cote d’Ivoire; Entebbe, Uganda; and Johannesburg, South Africa). The lab network sequences the 450 nucleotides coding for the carboxy-terminal 150 amino acids of the nucleoprotein. Data (as of March 29, 2017) are available from the Measles Nucleotide Surveillance database. http://www.who-measles.org/Public/Web_Front/main.php.

¶¶¶ Previous WHO recommendations from 2009 regarding the introduction of MCV2 required that national coverage of MCV1 be ≥80% for 3 consecutive years and that in the African region, at least one of the two main surveillance indicators be met for ≥2 years before introducing a second dose of measles vaccine into routine immunization.

**** Information on planning and implementing high-quality SIAs can be found at http://www.who.int/immunization/diseases/measles/en/.

†††† World Health Organization. African Regional Measles and Rubella Surveillance feedback summary for 2016. Data as of January 21, 2017.

§§§§ The WHO measles programmatic risk assessment tool (http://www.who.int/immunization/monitoring_surveillance/routine/measles_assessment/en) was developed to help national programs identify areas not meeting measles programmatic targets, and based on the findings, guide and strengthen measles elimination program activities and reduce the risk for outbreaks. This Excel-based tool assesses subnational programmatic risk as the sum of indicator scores in four categories: population immunity, surveillance quality, program performance, and threat assessment. Each subnational area is assigned to a programmatic risk category of low, medium, high, or very high risk based on the overall risk score. Scoring for each indicator was developed based on expert consensus.
